# Dose-dependent renoprotective effects of sacubitril/valsartan in heart failure: a retrospective study

**DOI:** 10.1080/0886022X.2025.2538830

**Published:** 2025-08-21

**Authors:** Takahiro Kato, Yusuke Nakano, Noriko Yasukawa, Masafumi Oonishi, Yukihiro Hamada

**Affiliations:** ^a^ Department of Pharmacy, Kochi Medical School Hospital, Kochi, Japan; ^b^ Department of Pharmacy, Aichi Medical University Hospital, Nagakute, Aichi, Japan; ^c^ Department of Cardiology, Aichi Medical University, Nagakute, Aichi, Japan; ^d^ AISAN Clinic, Tsushima, Aichi, Japan

**Keywords:** Heart failure, sacubitril/valsartan, renal protection, cardiorenal syndrome, angiotensin receptor neprilysin inhibitor, proteinuria

## Abstract

To evaluate the dose-dependent renoprotective effects of sacubitril/valsartan in heart failure patients. This retrospective observational study included patients with heart failure (Stage B or higher, B-type natriuretic peptide (BNP) >100 pg/mL or N-terminal proBNP >300 pg/mL) who initiated sacubitril/valsartan (SV) treatment. Patients were classified by final SV daily dose (50, 100, 200, or 400 mg) at 18 months. Factors associated with eGFR changes were identified using multiple regression analysis. A total of 157 patients (mean age 74.8–77.9 years, 64.3% male) were stratified by daily SV dosage groups (50 mg, *n* = 20; 100 mg, *n* = 46; 200 mg, *n* = 62; 400 mg, *n* = 29). Baseline characteristics were similar across groups for eGFR, heart failure stage, diabetes history, myocardial infarction, atrial fibrillation, proteinuria, and use of most heart failure medications. However, hypertension prevalence and systolic blood pressure differed significantly between groups (*p* < 0.05). One-way ANOVA revealed significant dose-dependent differences in eGFR changes among SV dosage groups (*p* < 0.05). In the final multiple linear regression model, SV dosage (*p* < 0.05) was a significant factor associated with eGFR changes, with proteinuria showing a trend toward significance. Sex and BNP levels ≥400 pg/dL were not significant. Sensitivity analysis converting SV dosage to a categorical variable confirmed these findings. Stratification by proteinuria status demonstrated dose-dependent relationships in both proteinuria-positive and proteinuria-negative subgroups, with more pronounced dose dependency in the proteinuria-positive group (*p* < 0.001). SV exhibits dose-dependent renoprotective effects in heart failure patients. Optimizing SV dosage may be beneficial for heart failure patients with concurrent kidney dysfunction, especially those with proteinuria.

## Introduction

Sacubitril/valsartan (SV), an angiotensin receptor-neprilysin inhibitor (ARNI), is a combination drug consisting of sacubitril and valsartan that holds a pivotal role in the treatment of heart failure. The PARADIGM-HF trial demonstrated that ARNI significantly reduces the risk of heart failure-related hospitalization and mortality in patients with heart failure with reduced ejection fraction (HFrEF), showing superior clinical efficacy over conventional angiotensin-converting enzyme (ACE) inhibitors or angiotensin II receptor blockers (ARBs) [1].

The pharmacological effects of SV are driven by its two main components. Sacubitril inhibits neprilysin, preventing the degradation of natriuretic peptides (NP) and increases their plasma levels [2]. While neprilysin inhibition alone can lead to higher plasma levels of angiotensin II, potentially causing adverse cardiovascular effects [3,4], the co-administration of valsartan mitigates this by blocking angiotensin II receptors. This combined mechanism enhances natriuresis, diuresis, and vasodilation while also suppressing the activity of the renin-angiotensin-aldosterone system (RAAS) and reducing sympathetic nervous system activity [2]. These combined effects suggest that SV not only protects the heart but also the kidneys.

Regarding renoprotective effects, *in vitro* and animal model studies have reported that ARNI provides superior renal protection compared to ACE inhibitors or ARBs [5–7]. Specifically, ARNI increases natriuretic peptide levels, which suppress podocyte damage and improve microcirculation in the kidneys. Furthermore, studies using diabetes mellitus (DM) and hypertension (HTN) model rats have demonstrated that ARNI exerts stronger renoprotective effects compared to ARBs [8].

A subanalysis of the PARADIGM-HF trial revealed that in heart failure patients with diabetes, SV significantly attenuates the decline in renal function compared to enalapril [9]. Moreover, systematic reviews and meta-analyses support the renoprotective effects of SV [10], highlighting its potential utility in heart failure patients.

SV has been reported to increase plasma levels of atrial natriuretic peptide (ANP) in a dose-dependent manner within hours after administration [11]. Observational studies in heart failure have shown that maintaining the maximum dose of SV, rather than reducing it, results in greater suppression of cardiovascular events [12,13]. This suggests a relationship between SV dosage and prognosis in heart failure patients. However, the relationship between SV dosage and renal function in heart failure patients remains unclear.

The aim of this study was to investigate the impact of SV dosage on renal function in heart failure patients, focusing on the renoprotective effects of ARNI. This study is expected to provide valuable insights into treatment strategies using SV for heart failure, particularly regarding renal protection.

## Methods

### Patients

This retrospective observational study included heart failure patients aged 18 years or older who were at Stage B or higher, had BNP levels greater than 100 pg/mL or NT-proBNP levels greater than 300 pg/mL, and initiated SV treatment at Aichi Medical University Hospital between August 2020 and December 2022. Patients were excluded if they discontinued SV during the study period, had a baseline eGFR of less than 20 mL/min/1.73 m^2^. Pregnant patients were also excluded. This study was conducted in accordance with the Declaration of Helsinki, and the study protocol was approved by the Institutional Review Board (approval number: 2023-254).

### Data collection

Data were retrospectively collected from electronic medical records. The initiation date of SV treatment was defined as the baseline, and eGFR was assessed during the 18-month follow-up. Observational data closest to the target time points within a range of ±3 months were utilized. For patients missing data at 18 months due to death or transfer after 12 months, the Last Observation Carried Forward method was applied for imputation. The dataset used for the final analysis comprised data available as of June 2024 from patients with accessible 18-month data at the time of the study.

### Dosage of sacubitril/valsartan

SV dosage for each patient was determined at the discretion of the attending clinicians. The study participants received treatment with one of the following daily doses of SV: 50 mg, 100 mg, 200 mg, or 400 mg, based on the available tablet strengths of 50 mg, 100 mg, and 200 mg. The SV dosage at 18 months was defined as the treatment dose for analysis, and patients were classified into groups based on these different dosage levels. The effects were compared across the dosage groups. The administration and dose escalation of SV were conducted in accordance with the Japanese package insert [14]. Specifically, SV was initiated at 50 mg twice daily (orally), with gradual escalation up to 200 mg per dose if tolerability was confirmed.

### Outcome

The primary endpoint was the change in estimated glomerular filtration rate (eGFR) from baseline (at the initiation of SV treatment) to 18 months. The change was calculated as the difference between the 18-month value and the baseline value, divided by the baseline. As for adverse events, we monitored changes in serum potassium levels and identified patients whose serum potassium levels exceeded 5.5 mmol/L.

### Statistical analysis

Based on the findings of a previous study [15], which reported a difference of 8 mL/min/1.73 m^2^ in eGFR change between the SV 400 mg/day group and the ACE inhibitor group, we assumed a dose-dependent effect. Specifically, the difference in eGFR change between the 100 mg/day and 400 mg/day groups was hypothesized to be 12%, with a standard deviation of 16. Finally, a sample size of at least 28 patients per group was calculated as necessary.

To minimize selection bias and reflect real-world effectiveness, all patients who were prescribed/initiated the study drug were included in the analysis, regardless of subsequent discontinuation, consistent with the principle of intention-to-treat.

For the primary endpoint, differences in eGFR change among the SV dosage groups were evaluated using one-way analysis of variance (ANOVA) to compare mean values across the groups. Additionally, Bonferroni correction was applied for multiple comparisons to address the issue of multiplicity. Statistical significance was set at *p* < 0.05, and all analyses were conducted using EZR software (version 1.68; Saitama Medical Center, Jichi Medical University, Saitama, Japan) [16].

Multiple linear regression analysis was performed to evaluate factors associated with changes in eGFR, accounting for patient background characteristics. Variables were selected for multivariate analysis based on the results of univariate analysis: continuous variables with a correlation coefficient of ≥ 0.2 with eGFR change and categorical variables with *p* < 0.2.

Sensitivity analyses were performed to assess the robustness of the results. Significant factors identified in the multiple regression analysis were reevaluated by converting continuous variables into categorical variables.

## Results

### Patients

**Figure 1.**
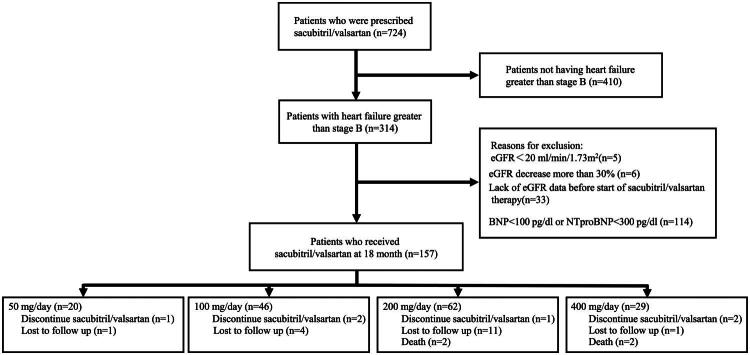
Study flowchart.

The study design and patient selection process are illustrated in Figure 1. A total of 157 patients were ultimately included in the analysis. The baseline characteristics of the study participants stratified by SV dosage groups are presented in Table 1. The cohort comprised 64.3% males, with a mean age ranging from 74.8 ± 11.9 to 77.9 ± 10.4 years across the groups. The distributions of eGFR, heart failure (Stage C), history of diabetes, myocardial infarction, atrial fibrillation, presence of proteinuria, and the use of heart failure medications, such as β-blockers and SGLT2 inhibitors, were similar across the dosage groups. However, there were statistically significant differences in the proportion of patients with hypertension and in systolic blood pressure among the groups (*p* < 0.05).

**Table 1.** Baseline characteristics of patients.

Factor	All patients (*n* = 157)	50 mg (*n* = 20)	100 mg (*n* = 46)	200 mg (*n* = 62)	400 mg (*n* = 29)	*p* value
Sex (Male; %)	101 (64.3)	11 (55.0)	28 (60.9)	41 (66.1)	21 (72.4)	0.591
Age	76.6 ± 11.4	74.8 ± 11.9	76.2 ± 13.7	76.7 ± 9.9	77.9 ± 10.4	0.817
Heart failure (stage C) (%)	95 (60.5)	14 (70.0)	29 (63.0)	34 (54.8)	18 (62.1)	0.628
Diabetes mellitus (%)	61 (38.9)	9 (45.0)	13 (28.3)	27 (43.5)	12 (41.4)	0.37
Myocardial infarction (%)	36 (22.9)	5 (25.0)	11 (23.9)	12 (19.4)	8 (27.6)	0.831
Hypertension (%)	100 (63.7)	9 (45.0)	22 (47.8)	49 (79.0)	20 (69.0)	0.002
Atrial fibrillation (%)	67 (42.7)	8 (40.0)	20 (43.5)	26 (41.9)	13 (44.8)	0.987
Co-administrated drug						
Beta-blocker (%)	119 (75.8)	18 (90.0)	38 (82.6)	40 (64.5)	23 (79.3)	0.049
MRA (%)	63 (40.1)	10 (50.0)	21 (45.7)	22 (35.5)	10 (34.5)	0.506
SGLT2 inhibitor (%)	53 (33.8)	7 (35.0)	15 (32.6)	19 (30.6)	12 (41.4)	0.786
Verisiguat (%)	1 (0.6)	0 (0.0)	0 (0.0)	0 (0.0)	1 (3.4)	0.266
Ivabradine (%)	4 (2.5)	1 (5.0)	1 (2.2)	2 (3.2)	0 (0.0)	0.674
Corticosteroids (%)	9 (5.7)	2 (10.0)	4 (8.7)	2 (3.2)	1 (3.4)	0.489
Loop diuretics (mg/day)*	5.1 ± 15.3	5.5 ± 8.9	9.1 ± 25.5	3.2 ± 7.4	2.4 ± 6.4	0.168
Proteinuria (%)	26 (26.5)	5 (29.4)	4 (13.8)	10 (28.6)	7 (41.2)	0.218
LVEF (%)	48.4 ± 15.7	42.4 ± 12.1	48.3 ± 15.8	49.5 ± 15.3	50.7 ± 18.4	0.305
<40 (%)	50 (31.8)	11 (57.9)	13 (33.3)	18 (31.6)	8 (30.8)	0.415
40–50 (%)	24 (15.3)	3 (15.8)	6 (15.4)	9 (15.8)	6 (23.1)	
≥50 (%)	67 (42.7)	5 (26.3)	20 (51.3)	30 (52.6)	12 (46.2)	
Systolic blood pressure (mmHg)	132.8 ± 22.5	125.8 ± 20.1	124.0 ± 19.6	138.4 ± 21.2	140.0 ± 25.9	0.001
eGFR (mL/min/1.73 m^2^)	52.3 ± 19.2	53.8 ± 15.9	51.4 ± 21.1	54.7 ± 19.9	47.3 ± 16.5	0.368
BNP (pg/dL)	327.8 ± 267.9	360.7 ± 311.4	394.7 ± 306.0	268.0 ± 288.9	327.4 ± 299.3	0.098
BNP ≥ 400 pg/dL (%)	39 (24.8)	7 (35.0)	15 (32.6)	9 (14.5)	8 (27.6)	0.100

*Furosemide equivalent.

MRA: mineralocorticoid receptor antagonist; SGLT2-I: sodium-glucose cotransporter 2 inhibitor; LVEF: left ventricular ejection fraction; eGFR: estimated glomerular filtration rate; BNP: brain natriuretic peptide.

### Outcome

**Figure 2.**
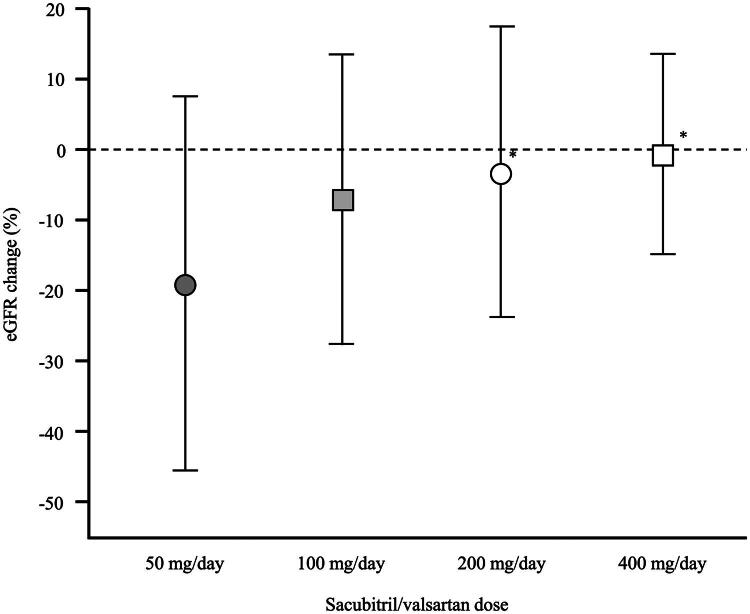
Changes in eGFR by sacubitril/valsartan dosage groups (50 mg/day, 100 mg/day, 200 mg/day, and 400 mg/day). Data are presented as mean ± standard deviation. *p < 0.05 vs. 50 mg/day group, analyzed using one-way ANOVA followed by Bonferroni-adjusted multiple comparisons.

The results of the one-way ANOVA indicated a significant difference in the change in eGFR among the SV dosage groups, demonstrating a dose-dependent relationship (*p* < 0.05). Furthermore, pairwise comparisons using the Bonferroni method revealed significant differences between the 50 and 200 mg groups, as well as between the 50 and 400 mg groups (Figure 2).

### Evaluation of dose dependency using multiple regression analysis

Univariate analysis identified factors potentially associated with changes in eGFR, including sex, BNP levels ≥ 400 pg/dL (*p* = 0.059), proteinuria positivity (*p* = 0.182), and SV dosage (r^2^ = 0.201, *p* < 0.05) (Table 2). The administration of SGLT2 inhibitors after 18 months did not have a significant impact. Multiple linear regression analysis of these factors revealed that SV dosage (*p* < 0.05) was a statistically significant factor associated with changes in eGFR, and proteinuria showed a trend toward significance (Table 3). Additionally, sensitivity analysis was conducted by converting the SV dosage from a continuous variable to a categorical variable. The results of the sensitivity analysis were consistent with the main findings.

**Table 2.** Univariate analysis of factors affecting eGFR changes from baseline.

	r2	*p* value
Age	−0.014	0.868
Sex		<0.05
Heart failure (stage C)		0.739
Diabetes mellitus		0.627
Myocardial infarction		0.302
Atrial fibrillation		0.924
Hypertension		0.386
Systolic blood pressure (mmHg)	−0.029	0.74
eGFR (mL/min/1.73 m^2^)	0.0148	0.865
BNP ≥ 400 pg/dL		0.059
LVEF (≤40%/41%–50%/50%≤)		0.438
Proteinuria		0.182
Sacubitril/valsartan dose (mg/day)	0.201	<0.05
Co-administrated drug		
Beta-blocker		0.312
MRA		0.782
SGLT2-I		0.448
Vericiguat		0.922
Ivabradine		0.958
Corticosteroids		0.925
SGLT2-I (at 18 months)		0.449
Loop diuretics (mg/day)*	0.036	0.671

*Furosemide equivalent.

eGFR: estimated glomerular filtration rate; BNP: brain natriuretic peptide; LVEF: left ventricular ejection fraction; MRA: mineralocorticoid receptor antagonist; SGLT2-I: sodium-glucose cotransporter 2 inhibitor.

**Table 3.** Results of multiple regression analysis for factors affecting eGFR changes.

	Estimated regression coefficient	95% CI	Standard error	*p* value
Sacubitril/valsartan dose (mg/day)	0.047	0.006–0.089	0.020	<0.05
Proteinuria	−10.7	−21.8–0.44	5.59	0.059
BNP ≧ 400 pg/dL	−6.66	−17.31–4.006	5.360	0.218
Sex (male)	8.14	−2.29–18.6	5.24	0.124

CI: confidence interval; BNP: brain natriuretic peptide.

### Association between proteinuria and changes in eGFR

**Figure 3.**
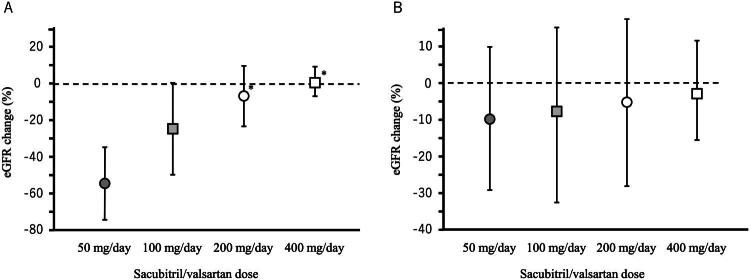
Changes in eGFR by sacubitril/valsartan dosage groups in patients with (A) and without (B) proteinuria. Data are presented as mean ± standard deviation. *p < 0.05 vs. 50 mg/day group, analyzed using one-way ANOVA followed by Bonferroni-adjusted multiple comparisons.

Patients were stratified based on the presence or absence of proteinuria, and the primary outcome—change in eGFR—was analyzed across different SV dosage groups using one-way ANOVA. The results indicated a dose-dependent relationship in both the proteinuria-positive and proteinuria-negative groups. Notably, in the proteinuria-positive group, statistically significant differences were observed between dosage groups (*p* < 0.001), with a more pronounced dose dependency (Figure 3).

### Serum potassium abnormalities

**Figure 4.**
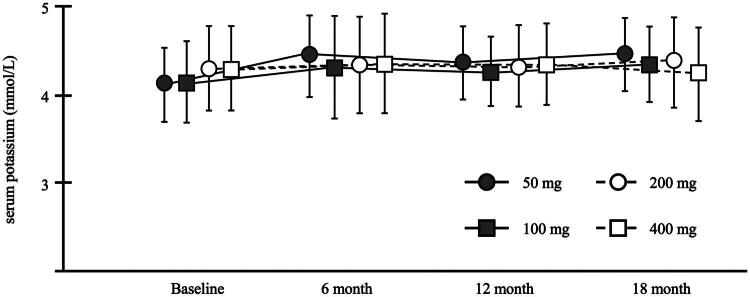
Serial changes of serum potassium. Data are presented as mean ± standard deviation. There was no significant difference analyzed using one-way ANOVA.

Changes in serum potassium levels showed no significant differences among all groups when analyzed using one-way ANOVA (Figure 4). The number of patients who experienced serum potassium levels ≥5.5 mmol/L during the study period was 7, 17, 13, and 9 in the 50 mg, 100 mg, 200 mg, and 400 mg groups, respectively (*p* = 0.66).

## Discussion

The results of this study suggest a dose-dependent relationship between the administration of SV and its renoprotective effects. Furthermore, this trend was particularly pronounced in patients with positive proteinuria, indicating that the dose-dependent renoprotective effects of SV may be more significant in this patient population.

A dose-dependent effect of SV is considered plausible. Although the present findings exhibit large standard deviations, and the rate of eGFR decline in the 400 mg group is significantly smaller compared to previously reported studies, the effect size may not be highly reliable. However, the observed eGFR changes across dose groups ranged from −0.25 to −9.5, which, considering the small sample size and findings from the PARADIGM-HF subanalysis [7] and retrospective study reports [15], appear to be within a reasonable range.

In this study, baseline blood pressure was not identified as a factor influencing changes in renal function. In a rat model of type 2 diabetes mellitus, even when blood pressure was adjusted to similar levels using hydralazine in the valsartan-treated group, the SV group demonstrated significantly suppressed elevations in serum creatinine and BUN, as well as proteinuria, at 76 weeks [7]. These findings suggest that the renoprotective effects of SV observed in this study may be influenced by mechanisms other than blood pressure.

SV increases blood ANP levels in a dose-dependent manner within a few hours of administration [11]. A study examining the prognosis of HFrEF based on dose adjustments of SV and enalapril reported that even when the dose of SV was reduced, it provided better outcomes compared to dose reductions of enalapril. However, patients who required dose reductions showed less benefit than those who did not [12]. Additionally, real-world data observing dose-dependent outcomes indicated that SV exerts dose-dependent effects in suppressing cardiovascular events [13]. The findings of our study align with these observations, suggesting that SV may also exhibit dose-dependent effects on renal function. In this study, we also monitored changes in serum potassium levels, but found no clear association between SV dosage and serum potassium values, suggesting that reverse causality in the relationship between renal function decline and SV dose escalation is unlikely.

The UK HARP-III trial did not demonstrate the superiority of SV at 400 mg/day over irbesartan at 300 mg/day for renoprotective effects in patients with chronic kidney disease (CKD) [17]. In that study, only 4% of participants had heart failure, and BNP levels were only mildly elevated. In contrast, our study focused on patients with heart failure and BNP levels exceeding 100 pg/mL or NT-proBNP levels exceeding 300 pg/mL. Based on our results, SV may exhibit renoprotective effects, particularly in patients with moderately elevated BNP levels.

Proteinuria is a risk factor for the progression of chronic CKD [18], and in this study, it was also identified as a factor associated with renal function deterioration. Importantly, the presence or absence of proteinuria influenced the dose-dependent renoprotective effects of SV. In this study, the dose-dependent renoprotective effects of SV were markedly observed in patients with positive proteinuria. Post-hoc analyses of randomized controlled trials have reported that while urinary protein levels increased in the SV group compared to the enalapril and valsartan groups, the decline in renal function was more gradual [19,20]. Close attention should be paid to changes in proteinuria following SV administration; however, in patients with positive proteinuria, active dose escalation of SV should be considered. Conversely, in the absence of proteinuria, the impact of SV on renal function may be minimal.

A target dose of 400 mg/day of SV is recommended to improve heart failure outcomes [12]. However, one of the adverse effects of SV is blood pressure reduction. This effect is also utilized in hypertension treatment, where SV produces a significantly greater blood pressure-lowering effect compared to valsartan monotherapy [21,22]. A network meta-analysis further suggested a dose-dependent blood pressure-lowering effect, showing a trend toward greater reductions at SV doses of 200 mg or higher compared to 100 mg [23].

In clinical practice, blood pressure reduction is often encountered as a dose-limiting factor when attempting to titrate SV in heart failure patients. Our previous investigation also demonstrated that blood pressure is particularly prone to decline in patients with a dehydration tendency [24]. Therefore, it is crucial to carefully assess the condition of each heart failure patient and determine the appropriate SV dose that maximizes its renoprotective effects. For patients with proteinuria who cannot tolerate higher SV doses due to blood pressure-related risks, efforts should be made to mitigate these risks as much as possible. For instance, reducing the dose of concomitant diuretics in patients with a dehydration tendency is one potential approach [24].

This study has several limitations. First, as a retrospective study, it was not possible to control for concomitant medications or eliminate biases arising from patient background factors during the study period. Sacubitril-valsartan has adverse effects, such as hypotension and hyperkalaemia, which are considered clinical dose-limiting factors. Therefore, factors identified as risk factors for these side effects [25], including age, left ventricular ejection fraction, eGFR, history of diabetes, history of hypertension, baseline blood pressure, and the use of mineralocorticoid receptor antagonists, beta-blockers, and steroids at the initiation of SV, were analyzed. Although patients in the high-dose group tended to have higher blood pressure, blood pressure was not selected as a factor contributing to renal function decline. This study lacked data on post-treatment blood pressure, limiting a thorough examination of its impact. However, since SV is suggested to exert dose-dependent blood pressure-lowering effects [23], higher blood pressure in certain patients may have positively influenced renal function outcomes. Second, this study was a small-scale, single-center study, and the influence of small sample size on the analysis could not be fully excluded. Diabetes is a known risk factor for renal function decline, and differences in the rate of eGFR decline between patients with and without diabetes have been reported among those treated with SV [9]. However, in this study, diabetes was not identified as a significant factor associated with the rate of eGFR decline. Third, the study lacked sufficient laboratory data necessary for comprehensive evaluation. Quantitative analysis of proteinuria was not adequately available, and assessments were based on qualitative analyses. Furthermore, while a Cox proportional hazards model was considered more appropriate for evaluating the dose-dependency of SV in this study, it was not performed due to the judgment that the sample size was insufficient. Finally, as SV dose adjustments were determined at the discretion of the prescribing physicians without a standardized protocol, the possibility of reverse causality in the relationship between renal function decline and SV dose escalation cannot be completely ruled out. Future studies addressing these limitations and providing further investigation are warranted.

In conclusion, this study provides valuable insights into the potential dose-dependent renoprotective effects of SV. In heart failure patients treated with SV, a dose-dependent renoprotective effect was suggested. Notably, this effect appeared to be more pronounced in patients with positive proteinuria. To further validate these findings, future prospective, multicenter studies with larger cohorts are warranted. In addition, investigation of the underlying mechanisms through biomarker analysis, evaluation of real-world data, and assessment of the potential synergistic effects of SGLT2 inhibitors may provide deeper insights into the renoprotective properties of SV. Long-term follow-up studies will also be crucial to determine its impact on CKD progression and the outcomes of renal replacement therapy.
